# Comparison of Early-Stage Mothers and Childless Women Seeking Pregnancy: Experienced Stress, Resilience and Satisfaction with Relationship with the Partner

**DOI:** 10.3390/ijerph18052543

**Published:** 2021-03-04

**Authors:** Alicja Malina, Dorota Suwalska-Barancewicz

**Affiliations:** Department of Social Psychology, Kazimierz Wielki University, 85-064 Bydgoszcz, Poland; dsbarancewicz@ukw.edu.pl

**Keywords:** motherhood, infertility, stress, quality of relationship

## Abstract

Introduction: The birth of a child is a significant turning point in the life of a woman. It is a source of personal fulfilment, but also a great challenge. On the other hand, the inability to conceive a child in a natural way results in a serious distortion of a woman’s psychic balance. Becoming a mother is an indicator of personal fulfilment and the source of social acceptation. Therefore, both becoming a mother and the inability to conceive a child in a natural way may be seen as stressful factors that impact a woman’s life. Aim of the study: The research project aimed to analyze the differences in stress, resilience and satisfaction with relationship and sexual satisfaction between early-stage mothers, i.e., women having a child and childless women seeking pregnancy. Sample: The study involved 80 women—40 mothers and 40 infertile women. The mean age of the mothers was 31.10 yrs (SD = 3.76) and of the childless women seeking pregnancy—31.48 yrs (SD = 3.35). Both groups were homogenous with regards to education, place of residence, the form and time of their relationship with partners. Method: The perceived stress was measured with the Perceived Stress Scale PSS—Polish adaptation by Juczyński and Ogińska-Bulik. Resilience was measured with Personal Adaptation Scale (LIRS-pl)—Polish adaptation by Malina, Pooley and Harms. The quality of the relationship was measured using the Quality of Relationship Inventory (QRI) adapted to Polish version by Suwalska-Barancewicz, Liberska and Izdebski and the sexual satisfaction was measured with the Sexual Satisfaction Questionnaire by Nomejko and Dolińska-Zygmunt. Results and conclusions: Statistical analyses indicate that the participating women differ in the level of perceived stress (higher among childless women seeking pregnancy; t = 2.721; *p* = 0.008), the depth/intensity of the relationship (higher among childless women seeking pregnancy; t = 2.376; *p* = 0.020) and conflict (higher among mothers; t = −2.829; *p* = 0.006). This research project proved that infertility and its consequences are not only negative. The research has shown that lower levels of relationship conflicts and greater commitment occur more frequently among infertile women than among mothers. Regardless of the observed differences in the studied sample, it is noteworthy that the compared groups are also similar to some extent. They neither differed in the level of personal resilience nor perceived support. Therefore, although the two experienced issues seem to be different, they may appear psychologically similar to some extent.

## 1. Introduction

The birth of a child is a very important turning point, particularly in the life of a woman. It introduces changes into the system of behaviors and experiences, both in the course of development [1] and in the assessment of the quality of life [2,3]. Motherhood can bring many positive but also negative experiences [4]. It can be seen as a source of self-fulfillment, or personal development [5].

On the other hand, having children is associated with a disturbance of the existing order in a woman’s life and the resulting emotional tension. The need to perform many tasks at the same time makes women overburdened with the roles they are trying to juggle. Duties such as housekeeping, upbringing and caring for children, often combined with professional activity, are demanding and become a source of tension and frustration [6]. Daily functioning is impeded by fatigue resulting from the lack of sleep and rest and a reduced amount of spare time for satisfying personal desires and needs. Relationships with particular family members, especially the partner, may also deteriorate [5].

The mechanisms of emotional regulation of couples struggling with the problem of infertility are also disrupted [7]. Long-term difficulties in becoming a parent have numerous negative consequences, and include: reduced self-esteem, low mood or stress. This is often associated with a lower appraisal of one’s physical attractiveness, a lack of trust in partner and reduced self-confidence, hope and sense of security [7]. The inability to conceive a child in a natural way brings about a serious mental imbalance, especially for the woman, for whom taking on the role of a mother is an important determinant of personal fulfillment and a source of social acceptation [8].

Various aspects of social negativity towards assisted reproductive technology can intensify the sense of loss, shame and social mismatch that often accompany infertility [9,10,11,12]. There is also a widespread belief that fertility problems are a stress factor that can influence early parental behavior, and therefore, potentially affect the psychosocial development of the child [13,14].

Numerous studies have shown that infertility is associated with lower satisfaction with sexual life and relationships with the partner due to focusing on the medical aspects of procreation [15,16,17] and an extensive interference in the intimacy of the treated couple [7]. This results in anxiety about losing intimacy [18]. Women display a higher level of concern about the stability of the relationship than their male partners. Moreover, they tend to have a less-positive opinion about their marriage and sexual life than men [19,20]. Similarly, early-stage mothers may experience lower sexual satisfaction and higher dissatisfaction with the relationship with their partner. This is due to focusing on the child and its needs [5]. In both early-stage mothers and childless women seeking pregnancy, a decline in the quality of the relationship or in the satisfaction with the relationship can be an additional source of stress. Infertility can lead to a serious emotional crisis, loss of self-confidence, increased stress and reduced mood [21,22]. Unsuccessful conception attempts cause heavy frustration at the psychological and existential level [8].

The challenges faced by modern mothers make the contemporary motherhood appear more difficult in comparison with the motherhood of the previous generation [23,24,25]. In addition to the need to perform both family and professional roles, it is particularly difficult and stressful for today’s mothers to create an image of excellence integrating motherhood and a professional career. The perfect, idealized motherhood adversely affects women’s mental health—it reduces self-esteem and increases the level of stress, anxiety and guilt [26].

It is worth mentioning that Poland has a specific view on a mother’s role in society. The Polish general public is characterized by relatively traditional values and beliefs. This is, to some extent, due to the dominant role of the Roman Catholic church in Poland, as well as the public television propaganda. Women in Poland are seen as guardians of the family warmth and comfort and their main social role is to bring up children [26,27]. Sociologists argue that the *main voices on infertility treatment or abortion are those of politicians and clergy who create a set of mostly false beliefs making parenting a socially sensitive issue related to poor knowledge and moral assessment*. Research shows that the level of religiosity, measured by the frequency of religious practices, has a significant impact on the attitude towards non-traditional parenting [26,27,28,29].

In view of the difficulties experienced by early-stage mothers and childless women seeking pregnancy, and the importance of the mother–child relationship during early childhood development, an analysis and comparison of the functioning of both groups of women seems valuable. Although the two experienced issues seem to be different, they may appear psychologically similar to some extent.

The literature indicates that both women with infertility and mothers struggle with stress and its consequences. Both being a mother and not being able to experience motherhood create emotional difficulties for women (stress, anxiety, insecurity). In both situations the problem lies in the sensitive issue of the social role of a woman as a mother and the expectations towards her. Young mothers experience role overload, overwhelming responsibility, fatigue, and deterioration of the quality of relationship with their partner. In turn, the inability to conceive a child in a natural way is a serious disturbance of the mental balance of a woman, as taking on the role of a mother is in many cases an indicator of personal fulfillment and a source of social acceptation [30]. Nevertheless, the nature of the similarities and differences in the psychological situation of early-stage mothers and childless women seeking pregnancy has not been well described.

Therefore, the current study aims to explore the following questions:

Research question 1: Do childless women seeking pregnancy and early-stage mothers experience different levels of stress and use different resilience resources?

Research question 2: Do childless women seeking pregnancy and early-stage mothers assess the quality of their relationship and the quality of their sexual life differently?

Research question 3: Can the quality of the intimate relationship (marriage, cohabitation with a heterosexual partner) of childless women seeking pregnancy and early-stage mothers be predicted by their resilience and sexual satisfaction?

## 2. Method

### 2.1. Aim of the Research Project

The aim of the research was to compare early-stage mothers and childless women seeking pregnancy in the following aspects:

- stress
- resilience
- the quality of the relationship (as determined by the perceived level of support, the depth of the relationship and conflict)
- sexual satisfaction

We also intended to identify resilience and sexual satisfaction as predictors of the relationship’s quality of early-stage mothers and childless women seeking pregnancy.

### 2.2. Measurements

In the present study, we chose to compare and contrast perceived stress and the resources to cope with it, as well as the indicators of the relationship’s quality in early-stage mothers and childless women seeking pregnancy. Although the literature indicates that both early-stage mothers and childless women seeking pregnancy suffer from stress and its consequences, the nature of differences or similarities in the way they perceive their situation has not been described.

For the purpose of this research, a survey was created to collect basic sociodemographic data (age, education, duration and form of relationship).

Perceived Stress is understood as a reaction to stressful events. The measurement consists of assessing one’s own life situation in terms of stress intensity, i.e., to what extent is the situation unpredictable, uncontrolled or excessively burdensome. Different symptoms of distress, resulting from the burden of events, undergo subjective evaluation.

In the current study, we chose to use The Perceived Stress Scale PSS-10 in the original version [31], as well as the Polish adaptation [32] intended to measure the perceived stress over the previous month. The method involves assessing the appropriateness of 10 questions concerning various subjective feelings connected with personal problems, events, behaviors and coping strategies on a 5-point scale (0—never, 4—very often). The questions describe situations perceived by an individual as being beyond his or her ability to cope (e.g., ‘*In the last month, how often have you been upset because of something that happened unexpectedly?*’, ‘*In the last month, how often have you felt that you were unable to control the important things in your life?*’). The overall score of the scale (after changing the scores in some questions) is the sum of all points, the probability distribution ranging from 0 to 40. The higher the score, the higher the level of perceived stress.

The reliability of the PSS-10 scale measured by the Cronbach’s alpha coefficient is 0.86 [32].

*Resilience*, understood as the concept responsible for: ‘the potential to exhibit resourcefulness by using available internal and external recourses in response to different contextual and developmental challenges’ [33] (p. 34) is considered here as a set of protective factors that help to maintain performance and well-being despite unfavorable conditions. It includes three aspects: personal attributes (PA), family support (F) and peer support (P). The Polish adaptation of the Lifespan Individual Resilience Scale(pl) (LIRS(pl)) under the working title *Skala Osobistej Adaptacyjności* was used to measure resilience. LIRS(pl) is a self-report tool consisting of 12 statements assigned to three scales, corresponding to the three components of resilience—personal attributes (‘I achieve what I set out to do’), support from family (‘My family gives me strength’) and support from peers (‘I have a strong bond with my friends’). The subject marks their answers on a 7-point scale (1—strongly disagree, 7—strongly agree). The tool produces an overall resilience result as well as a result for individual subscales. The reliability of particular subscales measured by the Cronbach’s alpha coefficient is satisfactory and was (0.85) for personal attributes, (0.93) for family support and (0.94) for peer support [34]. The temporal stability, estimated with the Pearson correlation coefficient, based on two measurements obtained at an interval of 4 weeks, was as follows: personal attributes 0.84, family support 0.93, peer support 0.90 [34].

The *Quality of Relationships* is determined by three indicators: the depth of the relationship, the intensity of interpersonal conflict and the strength of perceived support [35]. *Depth* (D) is determined by the degree of commitment and positive evaluation of the relationship. *Conflict* (C) is the intensity of anger and ambivalent feelings towards the partner. *Perceived Support* (S), in turn, is seen as the extent to which a loved one can be depended on to provide help in different situations [35]. The quality of the relationship was measured with the Quality of Relationships Inventory (QRI) (Polish adaptation: [36]). It is a self-report tool consisting of 23 items, with answers marked on a 4-point scale. The higher the score on a given scale, the higher the intensity of the properties measured by the individual factors. The Perceived Support Scale is made up of 7 statements (‘*To what extent can you count on this person to listen to you when you are angry with someone?*’), the Depth Scale consists of 6 statements (‘*How significant is this relationship in your life?*’), while the Conflict Scale contains 10 statements (‘*How much does this person want you to change?*’). The reliability of the particular subscales measured by the Cronbach’s alpha coefficient is satisfactory and is (0.79) for support, (0.88) for conflict and (0.71) for depth of the relationship [36].

Sexual satisfaction was assessed using the *Sexual Satisfaction Scale (SSS)*. Sexual satisfaction is understood as a cognitive-emotional response of the subject to their own sexual activity. It was measured using the Sexual Satisfaction Questionnaire by Agnieszka Nomejko and Grażyna Dolińska Zygmunt [37]. The tool consists of 10 items (e.g., ‘*Sex is a source of pleasure for me*’; ‘*My sexual life frustrates me*’; ‘*I find my sexual life fulfilling*’), evaluated by the respondents on a 4-level Likert scale (1—strongly agree; 4—strongly disagree). The result indicates the level of sexual satisfaction. The higher the score, the higher the satisfaction. The reliability of the method as measured by the Cronbach’s alpha coefficient is satisfactory and is 0.83 [37].

### 2.3. Procedure and Data Collection

The survey involving mothers and infertile women was carried out on-line in July and August 2019, by sending out an access link to an electronic form. The online instruction consisted of the aim of the study including criteria and information regarding ethical considerations. The participants who chose to take part were then redirected to the questionnaires. Both groups of participants were recruited in separate ways. The group of women seeking pregnancy was recruited by a gynecologist from a clinic in Poland. The doctor was willing to send the link to the questionnaire to 40 of her patients, all of whom filled it in. In the case of early-stage mothers, the link was sent out to parenting groups on Facebook platform by the researcher. Of note, 296 early-stage mothers filled out the questionnaires altogether. For the means of the comparison with the group of women seeking pregnancy, 40 random participants were drawn from the data set. The on-line survey was carried out in accordance with the guidelines of the International Test Commission [38] taking into account: (1) authentication of the identity of the test takers, (2) informing the respondents about the purpose, procedure and scope of the survey and (3) ethical principles of security and confidentiality of the collected results.

### 2.4. Data Analyses

The Student’s t-distribution test and multiple regression analysis were used to verify the relationships between the variables adopted in the research model. Statistical analyses were carried out in the Statistica 12.0 statistical software (TIBCO Software Inc. StatSoft Poland).

## 3. Results

The study involved 80 women—40 mothers and 40 infertile women.

The inclusion criteria in the case of early-stage mothers group was to be in the early adulthood period (under 40 [39]), have at least one child under the age of 3 (which is the usual age when children start kindergarten in Poland) and not to work professionally at the time of the study (as professional work is usually seen as an additional source of stress due to role overload [40]). The women seeking pregnancy group was determined by being qualified for the first in vitro procedure, not having miscarriages nor children from previous relationships.

The mean age of mothers was 31.10 yrs (SD = 3.76) and the mean age of infertile women was 31.48 yrs (SD = 3.35). The groups were homogeneous in terms of education, place of residence, form and duration of relationship between partners (infertile woman: 8.86 years; SD = 4.31; mothers: 8.91; SD = 4.51). Among infertile women, 87.5% were wives, 10% were engaged and one woman was in a cohabiting relationship; 90% of mothers were married, 3 were engaged and 1 was in cohabitation. The average age of children of the fertile women was 27 months (SD = 4.94) (see Table 1).

The descriptive statistics of the examined variables are presented in the tables below. (Table 2 and Table 3). Results for sexual satisfaction, perceived stress, perceived support and the depth of relationship were relatively high in both groups of participants.

The next step was to determine the differences between early-stage mothers and women seeking pregnancy. The Student’s t-distribution test was used for this purpose.

Statistical analysis (t-Student Test) indicates significant differences between the two compared groups of women:

- childless women seeking pregnancy were characterized by a higher level of perceived stress (av. of 23.63) than women with children (20.05; t = 2.721; *p* = 0.008)
- infertile women displayed lower level of conflict (av. of 18.28) than women who were mothers (exhibiting a higher level of conflict; av. of 21.93; t = −2.829; *p* = 0.006)
- a higher level of depth of relationship and commitment was characteristic of women without children (av. of 21.40) in comparison with mothers (av. of 20.03; t = 2.376; *p* = 0.020). The obtained results are shown in Table 4.

We then proceeded to identify predictors of the relationship quality among childless women seeking pregnancy and early-stage mothers. The quality of the relationship consists of three components [35]: perceived support, conflict and depth of relationship. Multiple regression analysis was applied.

It was observed that sexual satisfaction is the predictor of the quality of relationship of women seeking pregnancy in terms of perceived support (β = 0.46; *p* = 0.006; F(6.33) = 2.89; *p*^2^ = < 0.001; R^2^ = 0.34), and in terms of conflict (β = −0.40; *p* = 0.02; F(6.33) = 2.14; *p* = < 0.001; R^2^ = 0.28). The higher the sexual satisfaction, the higher the level of support perceived by the partner and the lower the intensity of relationship conflicts.

**Figure 1:**
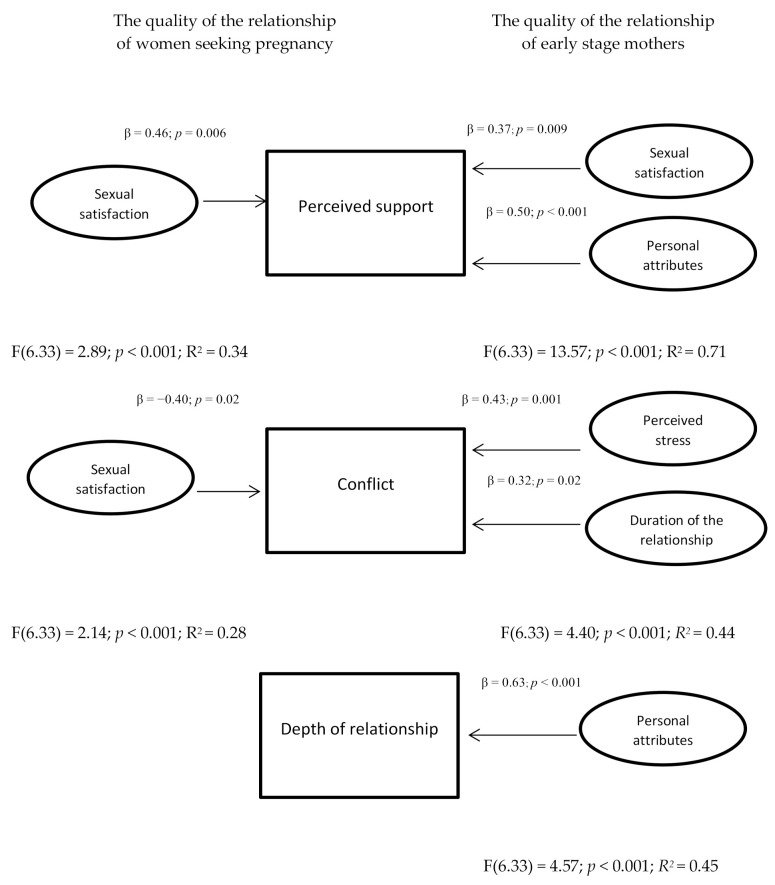
Comparison of predictors of quality of relationship between childless women seeking pregnancy and early-stage mothers.

The predictors of the quality of the relationship of early-stage mothers included the following: in terms of perceived support: sexual satisfaction (β = 0.37; *p* = 0.009); personal attributes (β = 0.50; *p* < 0.001; F(6.33) = 13.57; *p* < 0.001; R^2^ = 0.71). The higher the sexual satisfaction and the higher the assessment of one’s own coping attributes, the higher the level of perceived support from the partner; in terms of conflict: perceived stress (β = 0.43; *p* = 0.001); duration of the relationship (β = 0.32; *p* = 0.02 F(6.33) = 4.40; *p* < 0.001; R^2^ = 0.44). A higher level of perceived stress and a longer relationship was associated with a higher level of conflict in the relationship; in terms of the depth of the relationship: personal attributes (β = 0.63; *p* < 0.001 F(6.33) = 4.57; *p* < 0.001; R^2^ = 0.45). The higher the self-evaluation of the mothers’ personal attributes, the higher the depth of their relationship with their partner. The obtained results are shown in Figure 1.

## 4. Discussion

The research allowed for the identification of the similarities and differences in psychosocial functioning of childless women seeking pregnancy and women with offspring. It was proved that mothers and infertile women differ in terms of the perceived stress, the conflict in the relationship and the depth of the relationship (level of commitment).

Childless women seeking pregnancy were characterized by a higher level of perceived stress than women with children. This corresponds to the published reports, which emphasize that unintentional childlessness burdens both partners mentally. Women’s focus on family relationships causes them to develop chronic emotional tension [41]. Therefore, high levels of anxiety and tension make infertile women less stress-tolerant [42]. Results obtained in a Dutch sample showed that women experience lower satisfaction with life and are more prone to developing emotional problems during infertility treatment compared to their partners [43]. Furthermore, the negative effect of anxiety and tension is related to a lower assessment of their life quality [44].

Some studies show that stress levels among people affected by infertility depend on individual mental capacity and psychological qualities such as coping style and self-criticism. Passive coping strategies and a higher level of criticism are related to higher stress levels [45,46]. Moreover, self-intervention in cognitive coping and relaxation influence the emotional state and quality of life of women starting the invitro fertilization procedure (IVF). The application of this strategy leads to the improvement of the mental state of women (higher quality of life, lower level of anxiety, stress reduction) [47]. This present study did not confirm the occurrence of differences in resilience level among early-stage mothers and childless women seeking pregnancy. Nevertheless, one of the resilience’s dimensions—personal attributes—turned out to be a predictor of perceived partner-to-partner support among early-stage mothers.

Surprisingly, this research project proved that infertility and its consequences are not always negative. The research has shown that lower levels of relationship conflicts and greater commitment occur more frequently among infertile women than among mothers. Although the literature often raises the problem of increased conflicts among couples facing procreation difficulties, it turns out that this situation can be an opportunity to find greater closeness and intimacy, increase mutual trust and the sense of security and support [8,48]. What is more, an increase in conflicts and less commitment in the case of early-stage mothers may result from the overload of roles which they try to cope with. In some relationships, the woman is receiving little support from her partner, which leads to dissatisfaction with the relationship and can also be a source of conflicts and even mental problems [6,49,50,51]. The hypothesis that experiencing infertility could in some cases bring about positive outcomes was supported by studies conducted in Iran. The comparison of fertile and infertile couples indicated that fertile couples evaluate their quality of life higher, but they assess their sexual and marital satisfaction lower than infertile couples [52]. This was explained by potential financial problems and disturbance in couple’s relationships due to having children.

Regardless of the observed differences in the studied sample, it is noteworthy that the compared groups are also similar to some extent. They did not differ in the level of personal resilience nor perceived support. This could be due to the fact that in both cases—among early-stage mothers and women seeking pregnancy—the main source of support is the partner who is experiencing similar difficulties [53]. Also, in both groups we identified sexual satisfaction as one of the predictors of perceived partner support. This proves that satisfactory sexual life is, in general, the basis for maintaining a relationship [54,55,56], and at the same time strengthens partnership [57]. Considering the aforementioned, one of the important conclusions from the present study is that regardless of the source of stress or life crisis (e.g., early parenting or infertility stress), the partner’s relationship (perceived support, sexual fulfillment) plays a crucial role in maintaining good mental condition of women [54,58,59]. As mentioned above, this research also allowed for the identification of factors determining the quality of relationship of childless women seeking pregnancy and early-stage mothers. In the case of women experiencing procreative problems, the variable that significantly predicts the quality of the relationship in terms of perceived support from the partner and the level of conflicts in the relationship is sexual satisfaction. A higher level of sexual satisfaction results in a higher level of perceived support, and a lower level of satisfaction sets a higher level of conflict. In this present study, the perceived support from an intimate partner was defined as the degree to which the partners may rely on each other in different life situations [35]. The obtained results correspond to the results of other studies, which emphasize that the quality of sexual life in the face of procreation problems translates into the quality of functioning in the relationship. Scheduled intercourse disrupts intimacy and closeness between partners, generating further conflicts [18,43]. The analysis of articles on the influence of infertility on couples also confirms that infertility negatively affects the mental health, well-being and sexual satisfaction of partners [60].

Sexual satisfaction proved to be important also for women with children. Its high level was associated with higher perceived support from the partner. This shows the importance of a satisfactory sexual life for a happy marriage [57,61]. Many studies have shown that sexual satisfaction effectively diversifies satisfied and dissatisfied relationships—couples with higher levels of sexual satisfaction were more content [62,63]. In contrast, low satisfaction with sexual life adversely affects relationships, bonds with the partner, self-esteem, performance at work and family relations [64]. Nevertheless, it is worth mentioning that the results of sexual satisfaction in the studied sample were above the norm provided by the authors of the Sexual Satisfaction Scale [37]. Therefore, it may be assumed that the results are to some extent biased due to social desirability.

Resilience personal attributes used by an individual proved to be predictors of the quality of the early-stage mothers’ relationship in terms of perceived support and the depth of the relationship. The higher the mothers evaluated their own attributes for dealing with crisis, the higher level of their perceived support from partners, and the greater commitment. According to the definition provided by the authors of the tool, personal attributes refer to having a personal purpose in life, a positive outlook on the future, a sense of control, self-confidence and perseverance [65]. Therefore, if a woman has a positive view of her own attributes, she is also likely to have a positive outlook on the future. Consequently, she hopes that any parenthood-related difficulties can be quickly resolved. This may also involve a better perception of the relationship with the partner.

The final aspect of the quality of the relationship of early-stage mothers, conflict, was determined by the stress experienced by mothers and the duration of their relationship with partner. The higher evaluation of the women’s felt stress and the longer duration of their relationship was parallel to the higher intensity of the conflict. Some researchers believe that life stress experienced by couples plays a part in the etiology of conflict. Stressed partners generate more negative emotions in the relationship and show negative behavior when solving relationship-related problems. Less-stressed couples, on the other hand, are more likely to make attempts to work out negative behaviors towards the partner so that they can terminate conflict situations more quickly [66].

Short-term consequences of stress manifest themselves in emotions and changes in the physiological condition, while long-term consequences affect physical health, well-being and mental resilience or social functioning [67]. Moreover, stress factors adversely affect the durability [68] and quality of marriage [69]. Experiencing stress disrupts the hormonal, neural and mental balance of a person, which results in different patterns of behavior. How stress is dealt with depends on the strategy chosen—proactive and take-charge strategies are usually more effective. Partners who solve conflicts in a constructive way, employing positive communication and fewer negative interactions in particular, build relationships which are full of acceptance, intimacy and self-esteem [70].

The research showed that, in the case of mothers, the duration of the relationship is a predictor of conflicts in the relationship. This suggests negative changes for the marriage relations in time. This corresponds with the results obtained by Mason and Blankenship [71], which showed that a longer duration of the relationship was conducive to an increase in mental and physical abuse towards partners and a decrease in mutual commitment. Additionally, the results of a study by Kouros, Papp and Cummings [72] highlight the negative impact of the duration of the relationship on the interaction between the partners. Partners were also more prone to depressive symptoms as the relationship lasted. At this point, it seems reasonable to quote the results of a study by Rusbult [73], referring to the investment model in the context of commitment dynamics. The researcher claims that the connection between the partners is initially associated with positive rather than negative events in the relationship and the related satisfaction. This is due, among other things, to the partners’ desire to show their best side at the beginning of their relationship. As the relationship advances, the partners focus on negative rather than positive events and, as a result, report a decrease in satisfaction with life as costs increase. Moreover, as the relationship develops, the true features of the partners are revealed, the partner as well as the relationship are no longer romanticized, and more and more attention is paid to the costs of remaining in the relationship [74].

## 5. Limitations

The authors believe that the two main research points of this study are identifying marital relations as crucial when different kinds of adversities occur in life and finding positive aspects of partners relationship when facing fertility issues. The presented research, despite its significant scientific value, also has some limitations. One such limitation concerns the possibility of generalizing the research results determined with a relatively small sample. Nevertheless, it is worth mentioning that studies regarding fertility issues among Polish couples are hard to conduct due to their unwillingness to discuss problems openly. Also, the fact that early-stage mothers were recruited via parental forums may suggest that they were highly motivated and focused on parenting, or suffered from some difficulties that they could not cope with. Another issue is that we did not control the number of children that the early-stage mothers had. Having more children and the parenting experience could have affected their perception of their current situation. Nevertheless, we are assuming that stress and its consequences may have only been reduced—not boosted by previous parenting experiences. We also did not control the time elapsed from the beginning of infertility treatment, which may have affected experienced stress and the quality of relationship. The effect was to be minimalized by the fact that recruited women were qualified for their first in vitro procedure assuming that they had already had similar stress experiences beforehand as there is a standardized medical procedure related to qualifying for in vitro. According to these standards, all the couples that have not conceived after one year of unprotected vaginal sexual intercourse in the absence of any known cause of infertility are first qualified for 3–6 cycles of intrauterine insemination (IUI) and in the end who have not conceived during IUI cycles to in vitro fertilization.

Furthermore, the level of distribution of studied variables in the population of voluntary childless women is unknown. It would also be worth incorporating men’s perspective of parenthood and hindered procreation. This could provide more precise information on the nature of couples’ life satisfaction, as well as the quality and complexity of their relationship. In future research, it could also be beneficial to include other variables in the area of exploration relevant for the quality of relationship, motherhood and quality of life in general. The literature indicates that assessment of parental satisfaction is related to satisfaction with relationships [75,76], but also parental roles [27], stress management strategies, control over emotions [77], social support [48,78,79] and attachment style [80,81]. There are also measurements that are specific for diagnosing infertility-related stress that would be worth incorporating in future research [82]. Also, both women seeking pregnancy and early-stage mothers could have been controlled for preexisting conditions such as depression and anxiety disorders. In future research, it would be worthwhile to analyze the meaning of emotional and mental condition of women as, according to the literature, they may affect women’s life satisfaction and relationship quality [26,83].

In the search for determinants of the functioning of early-stage mothers and childless women seeking pregnancy in the dynamically changing world, one can additionally take into account external conditions important from the point of view of the quality of life, such as housing situation, financial support and division of household duties and the way they are performed, since, as the literature indicates, these are potentially conflicting factors which may affect different aspects of the quality of life assessment [6].

The research described does not exhaustively examine the broad spectrum of issues related to the hardships of motherhood, the challenges of women in the face of procreation problems, as it is only one of the many ways of exploring this issue. The presented results may serve as a starting point for further questions and further exploration in the area of family life quality.

## Figures and Tables

**Table 1:** Characteristics of the respondents: percentage distribution of sociodemographic variables in the sample.

Variable	Mothers %	Infertile Women %
**Education**		
Primary education	0	0
Secondary education	22.5	20
Higher education	77.5	80
**Place of Residence**		
Small village	7.5	10
City under 100,000 inhabitants	17.5	15
City between 100,000 and 500,000 inhabitants	52.5	47.5
City over 500,000 inhabitants	22.5	27.5
**Form of Relationship**		
Married	90	87.5
Engaged	7.5	10
Cohabitation	2.5	2.5

Source: own elaboration.

**Table 2:** Distribution of results (descriptive statistics)—women seeking pregnancy.

Variable	Descriptive Statistics: Infertile Women				
	N	Average	Minimum	Maximum	SD
Sexual satisfaction	40	30.63	15.00	40.00	5.91
Perceived stress	40	23.63	15.00	33.00	4.56
Personal attributes	40	21.93	10.00	28.00	3.31
Family	40	23.45	6.00	28.00	5.71
Friends	40	19.33	4.00	28.00	5.91
Total resilience	40	64.70	21.00	83.00	12.78
Perceived support	40	24.58	13.00	28.00	3.32
Conflict	40	18.28	9.00	30.00	4.93
Depth of relationship	40	21.40	13.00	24.00	2.13

Source: own elaboration.

**Table 3:** Distribution of results (descriptive statistics)—early- stage mothers.

Variable	Descriptive Statistics: Mothers				
	N	Average	Minimum	Maximum	SD
Sexual satisfaction	40	31.60	10.00	40.00	6.77
Perceived stress	40	20.05	4.00	36.00	6.95
Personal attributes	40	21.50	10.00	28.00	4.25
Family	40	23.96	6.00	28.00	4.99
Friends	40	19.50	4.00	28.00	5.91
Total resilience	40	64.98	37.00	83.00	11.96
Perceived support	40	23.83	7.00	28.00	4.17
Conflict	40	21.93	10.00	40.00	6.51
Depth of relationship	40	20.03	11.00	24.00	2.97

Source: own elaboration.

**Table 4:** Comparison of infertile women and mothers.

Variable	Comparison of Infertile Women and Mothers							
	Infertile Women (0)	Mothers (1)	*T*	*p*	N (0)	N (1)	SD (0)	SD (1)
Sexual satisfaction	30.63	31.60	−0.682	0.495	40	40	5.91	6.77
Perceived stress	23.63	20.05	2.721	0.008	40	40	4.56	6.95
Personal attributes	21.93	21.50	0.499	0.619	40	40	3.31	4.25
Family	23.45	23.98	−0.438	0.663	40	40	5.71	5.00
Friends	19.33	19.50	−0.132	0.895	40	40	5.91	5.91
Total resilience	64.70	64.98	−0.099	0.921	40	40	12.77	11.96
Perceived support	24.58	23.88	0.889	0.377	40	40	3.32	4.17
Conflict	18.28	21.93	−2.829	0.006	40	40	4.93	6.51
Depth of relationship	21.40	20.03	2.376	0.020	40	40	2.13	2.97

Source: own elaboration.

## Data Availability

The data presented in this study are available on request from the corresponding author. The data are not publicly available due to privacy.
